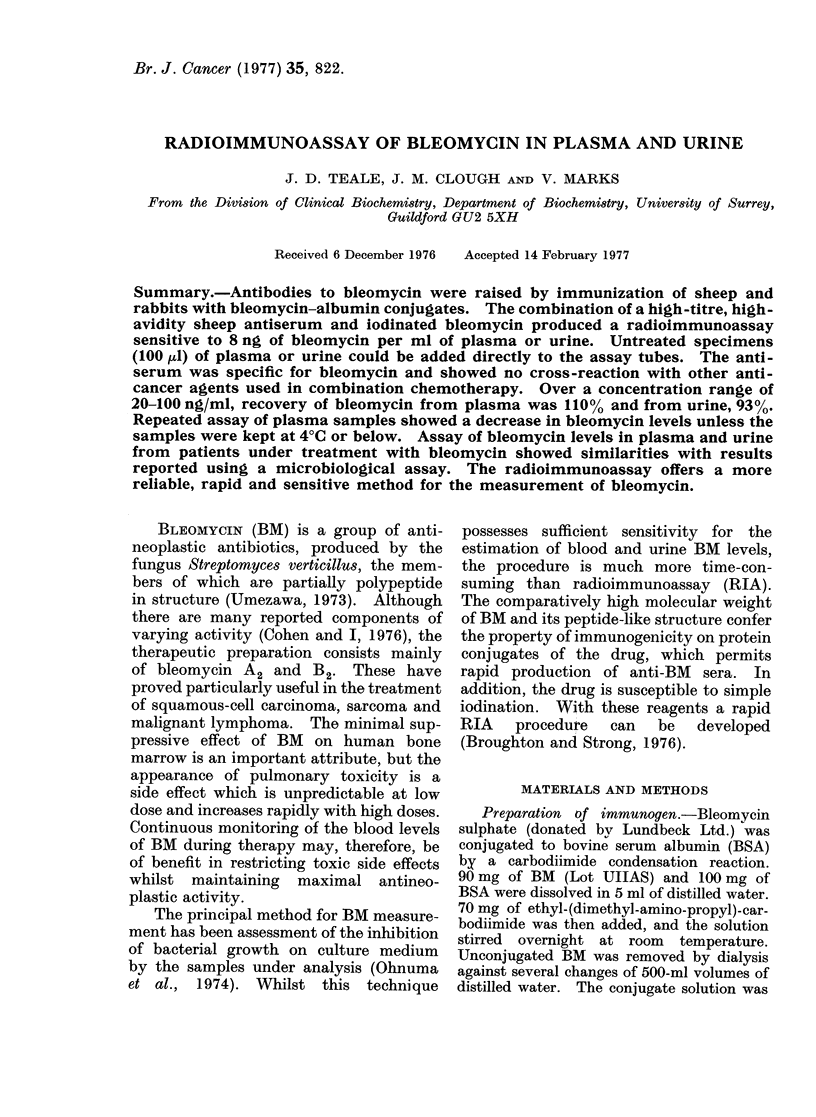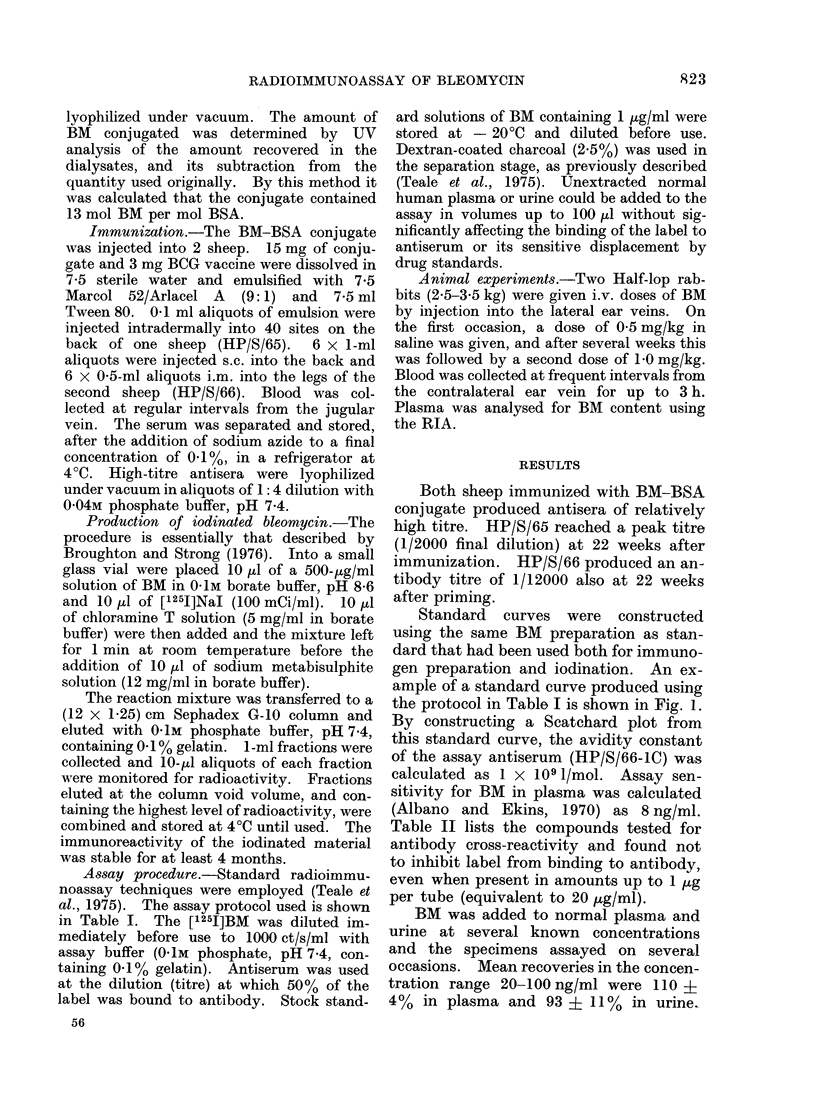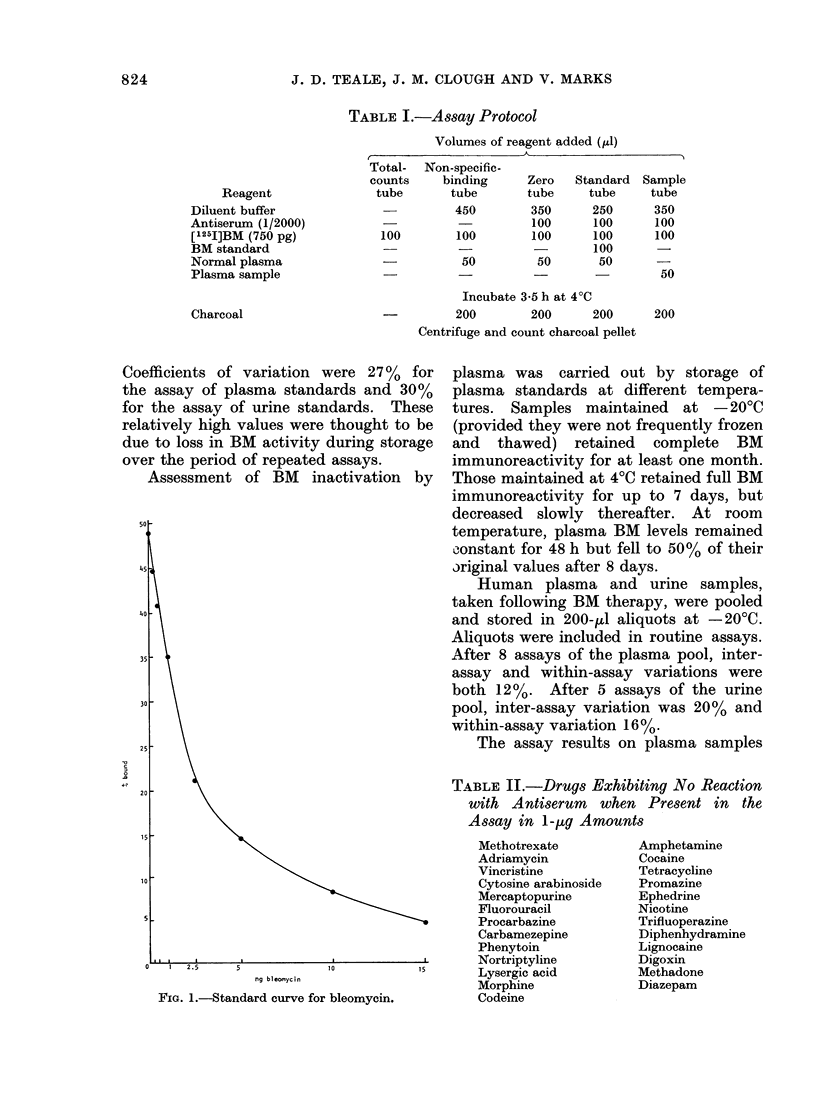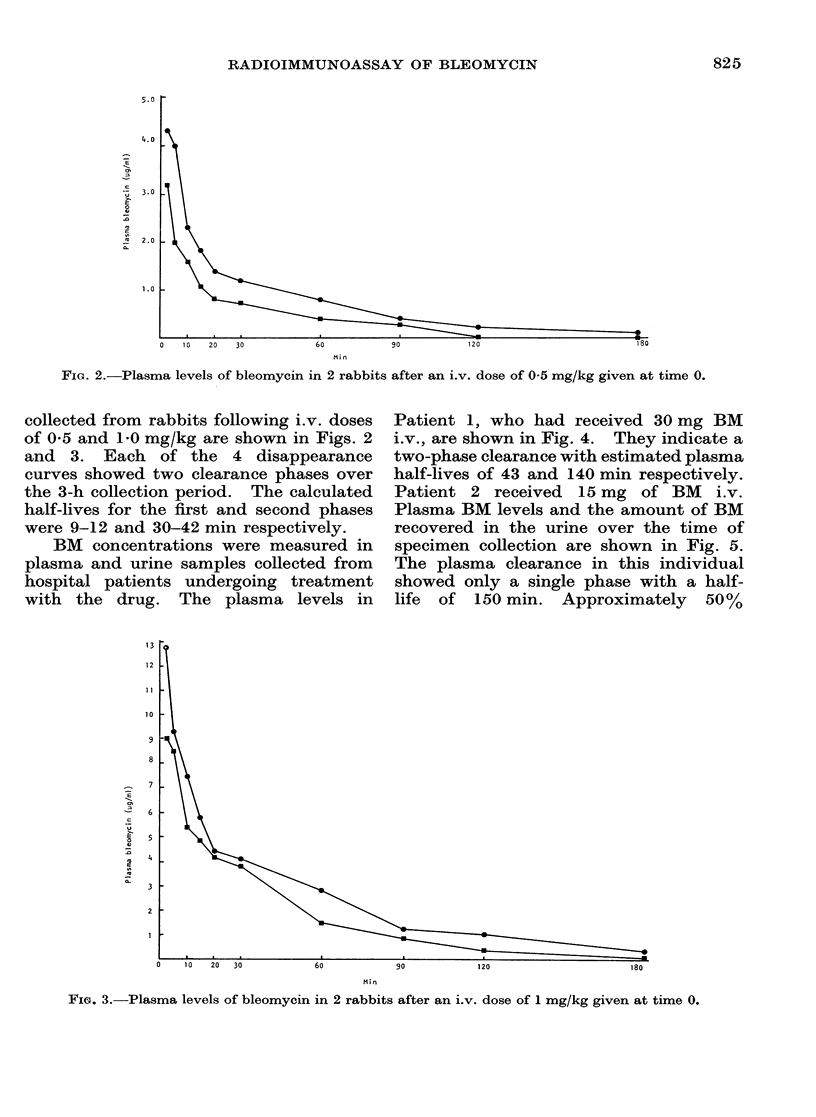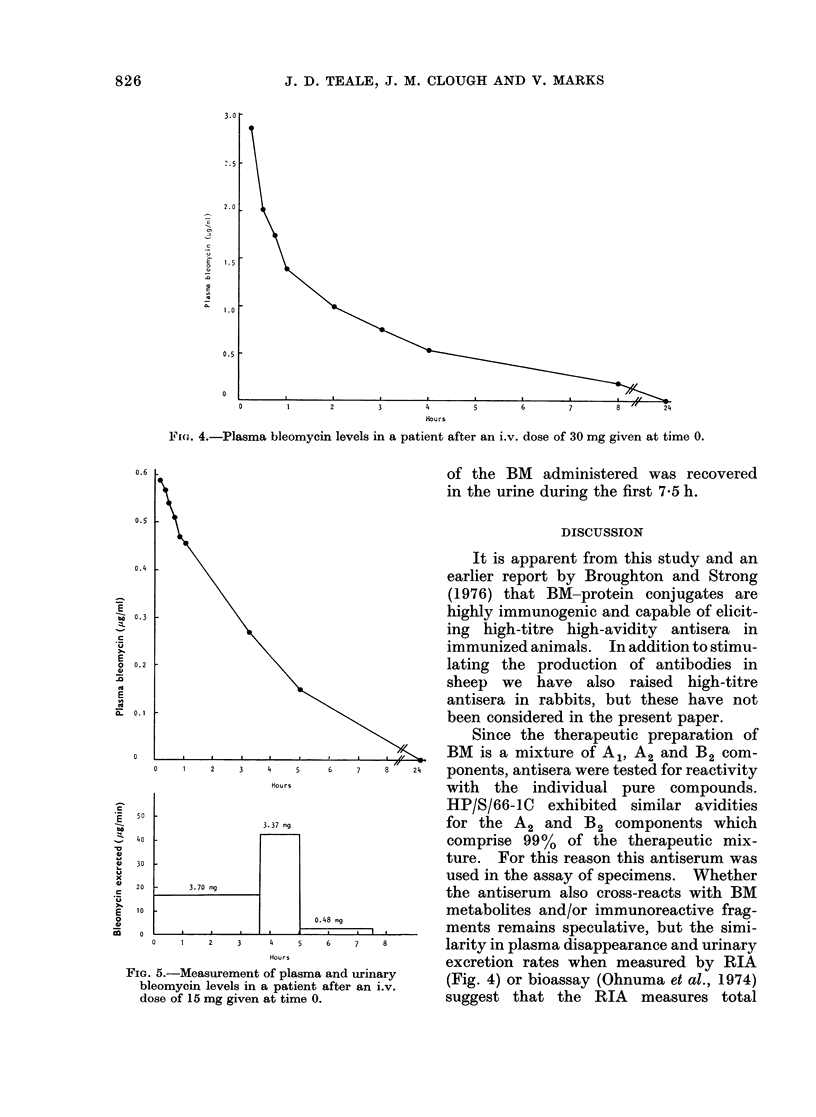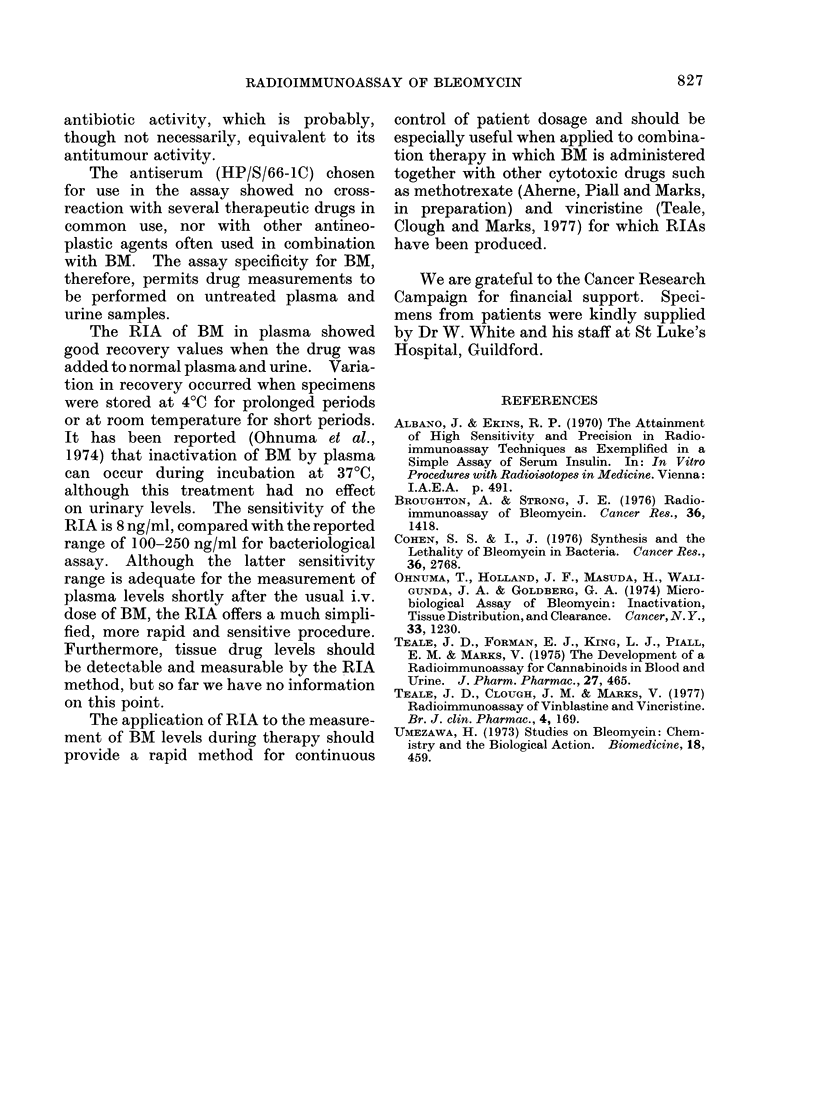# Radioimmunoassay of bleomycin in plasma and urine.

**DOI:** 10.1038/bjc.1977.124

**Published:** 1977-06

**Authors:** J. D. Teale, J. M. Clough, V. Marks

## Abstract

Antibodies to bleomycin were raised by immunization of sheep and rabbits with bleomycin-albumin conjugates. The combination of a high-titre, high-avidity sheep antiserum and iodinated bleomycin produced a radioimmunoassay sensitive to 8 ng of bleomycin per ml of plasma or urine. Untreated specimens (100 microliter) of plasma or urine could be added directly to the assay tubes. The antiseerum was specific for bleomycin and showed no cross-reaction with other anticancer agents used in combination chemotherapy. Over a concentration range of 20-100 ng/ml, recovery of bleomycin from plasma was 110% and from urine, 93%. Repeated assay of plasma samples showed a decrease in bleomycin levels unless the samples were kept at 4 degrees C or below.Assay of bleomycin levels in plasma and urine from patients under treatment with bleomycin showed similarities with results reported using a microbiological assay. The radioimmunoassay offers a more reliable, rapid and sensitive method for the measurement of bleomycin.


Br. J. Cancer (1977) 35, 822.

### RADIOIMMUNOASSAY OF BLEOMYCIN IN PLASMA AND URINE

J. D. TEALE, J. M. CLOUGH AND V. MARKS

*From the Division of Clinical Biochemistry, Department of Biochemistry, University of Surrey, Guildford GU2 5XH*

Received 6 December 1976 Accepted 14 February 1977

**Summary.—Antibodies to bleomycin were raised by immunization of sheep and rabbits with bleomycin–albumin conjugates. The combination of a high-titre, high-avidity sheep antiserum and iodinated bleomycin produced a radioimmunoassay sensitive to 8 ng of bleomycin per ml of plasma or urine. Untreated specimens (100 μl) of plasma or urine could be added directly to the assay tubes. The antiserum was specific for bleomycin and showed no cross-reaction with other anticancer agents used in combination chemotherapy. Over a concentration range of 20–100 ng/ml, recovery of bleomycin from plasma was 110% and from urine, 93%. Repeated assay of plasma samples showed a decrease in bleomycin levels unless the samples were kept at 4°C or below. Assay of bleomycin levels in plasma and urine from patients under treatment with bleomycin showed similarities with results reported using a microbiological assay. The radioimmunoassay offers a more reliable, rapid and sensitive method for the measurement of bleomycin.**

BLEOMYCIN (BM) is a group of antineoplastic antibiotics, produced by the fungus *Streptomyces verticillus*, the members of which are partially polypeptide in structure (Umezawa, 1973). Although there are many reported components of varying activity (Cohen and I, 1976), the therapeutic preparation consists mainly of bleomycin $A_2$ and $B_2$. These have proved particularly useful in the treatment of squamous-cell carcinoma, sarcoma and malignant lymphoma. The minimal suppressive effect of BM on human bone marrow is an important attribute, but the appearance of pulmonary toxicity is a side effect which is unpredictable at low dose and increases rapidly with high doses. Continuous monitoring of the blood levels of BM during therapy may, therefore, be of benefit in restricting toxic side effects whilst maintaining maximal antineoplastic activity.

The principal method for BM measurement has been assessment of the inhibition of bacterial growth on culture medium by the samples under analysis (Ohnuma *et al.*, 1974). Whilst this technique possesses sufficient sensitivity for the estimation of blood and urine BM levels, the procedure is much more time-consuming than radioimmunoassay (RIA). The comparatively high molecular weight of BM and its peptide-like structure confer the property of immunogenicity on protein conjugates of the drug, which permits rapid production of anti-BM sera. In addition, the drug is susceptible to simple iodination. With these reagents a rapid RIA procedure can be developed (Broughton and Strong, 1976).

### MATERIALS AND METHODS

*Preparation of immunogen.*—Bleomycin sulphate (donated by Lundbeck Ltd.) was conjugated to bovine serum albumin (BSA) by a carbodiimide condensation reaction. 90 mg of BM (Lot UIIAS) and 100 mg of BSA were dissolved in 5 ml of distilled water. 70 mg of ethyl-(dimethyl-amino-propyl)-carbodiimide was then added, and the solution stirred overnight at room temperature. Unconjugated BM was removed by dialysis against several changes of 500-ml volumes of distilled water. The conjugate solution was lyophilized under vacuum. The amount of BM conjugated was determined by UV analysis of the amount recovered in the dialysates, and its subtraction from the quantity used originally. By this method it was calculated that the conjugate contained 13 mol BM per mol BSA.

*Immunization.*—The BM–BSA conjugate was injected into 2 sheep. 15 mg of conjugate and 3 mg BCG vaccine were dissolved in 7·5 sterile water and emulsified with 7·5 Marcol 52/Arlacel A (9:1) and 7·5 ml Tween 80. 0·1 ml aliquots of emulsion were injected intradermally into 40 sites on the back of one sheep (HP/S/65). 6 × 1-ml aliquots were injected s.c. into the back and 6 × 0·5-ml aliquots i.m. into the legs of the second sheep (HP/S/66). Blood was collected at regular intervals from the jugular vein. The serum was separated and stored, after the addition of sodium azide to a final concentration of 0·1%, in a refrigerator at 4°C. High-titre antisera were lyophilized under vacuum in aliquots of 1:4 dilution with 0·04M phosphate buffer, pH 7·4.

*Production of iodinated bleomycin.*—The procedure is essentially that described by Broughton and Strong (1976). Into a small glass vial were placed 10 μl of a 500-μg/ml solution of BM in 0·1M borate buffer, pH 8·6 and 10 μl of [$^{125}$I]NaI (100 mCi/ml). 10 μl of chloramine T solution (5 mg/ml in borate buffer) were then added and the mixture left for 1 min at room temperature before the addition of 10 μl of sodium metabisulphite solution (12 mg/ml in borate buffer).

The reaction mixture was transferred to a (12 × 1·25) cm Sephadex G-10 column and eluted with 0·1M phosphate buffer, pH 7·4, containing 0·1% gelatin. 1-ml fractions were collected and 10-μl aliquots of each fraction were monitored for radioactivity. Fractions eluted at the column void volume, and containing the highest level of radioactivity, were combined and stored at 4°C until used. The immunoreactivity of the iodinated material was stable for at least 4 months.

*Assay procedure.*—Standard radioimmunoassay techniques were employed (Teale *et al.*, 1975). The assay protocol used is shown in Table I. The [$^{125}$I]BM was diluted immediately before use to 1000 ct/s/ml with assay buffer (0·1M phosphate, pH 7·4, containing 0·1% gelatin). Antiserum was used at the dilution (titre) at which 50% of the label was bound to antibody. Stock standard solutions of BM containing 1 μg/ml were stored at − 20°C and diluted before use. Dextran-coated charcoal (2·5%) was used in the separation stage, as previously described (Teale *et al.*, 1975). Unextracted normal human plasma or urine could be added to the assay in volumes up to 100 μl without significantly affecting the binding of the label to antiserum or its sensitive displacement by drug standards.

*Animal experiments.*—Two Half-lop rabbits (2·5–3·5 kg) were given i.v. doses of BM by injection into the lateral ear veins. On the first occasion, a dose of 0·5 mg/kg in saline was given, and after several weeks this was followed by a second dose of 1·0 mg/kg. Blood was collected at frequent intervals from the contralateral ear vein for up to 3 h. Plasma was analysed for BM content using the RIA.

### RESULTS

Both sheep immunized with BM–BSA conjugate produced antisera of relatively high titre. HP/S/65 reached a peak titre (1/2000 final dilution) at 22 weeks after immunization. HP/S/66 produced an antibody titre of 1/12000 also at 22 weeks after priming.

Standard curves were constructed using the same BM preparation as standard that had been used both for immunogen preparation and iodination. An example of a standard curve produced using the protocol in Table I is shown in Fig. 1. By constructing a Scatchard plot from this standard curve, the avidity constant of the assay antiserum (HP/S/66-1C) was calculated as 1 × 10$^{9}$ l/mol. Assay sensitivity for BM in plasma was calculated (Albano and Ekins, 1970) as 8 ng/ml. Table II lists the compounds tested for antibody cross-reactivity and found not to inhibit label from binding to antibody, even when present in amounts up to 1 μg per tube (equivalent to 20 μg/ml).

BM was added to normal plasma and urine at several known concentrations and the specimens assayed on several occasions. Mean recoveries in the concentration range 20–100 ng/ml were 110 ± 4% in plasma and 93 ± 11% in urine.

TABLE I.—*Assay Protocol*

| | Volumes of reagent added (μl) | | | | |
|---|---|---|---|---|---|
| Reagent | Total-counts tube | Non-specific-binding tube | Zero tube | Standard tube | Sample tube |
| Diluent buffer | — | 450 | 350 | 250 | 350 |
| Antiserum (1/2000) | — | — | 100 | 100 | 100 |
| [$^{125}$I]BM (750 pg) | 100 | 100 | 100 | 100 | 100 |
| BM standard | — | — | — | 100 | — |
| Normal plasma | — | 50 | 50 | 50 | — |
| Plasma sample | — | — | — | — | 50 |
| | | Incubate 3·5 h at 4°C | | | |
| Charcoal | — | 200 | 200 | 200 | 200 |
| | | Centrifuge and count charcoal pellet | | | |

Coefficients of variation were 27% for the assay of plasma standards and 30% for the assay of urine standards. These relatively high values were thought to be due to loss in BM activity during storage over the period of repeated assays.

Assessment of BM inactivation by plasma was carried out by storage of plasma standards at different temperatures. Samples maintained at —20°C (provided they were not frequently frozen and thawed) retained complete BM immunoreactivity for at least one month. Those maintained at 4°C retained full BM immunoreactivity for up to 7 days, but decreased slowly thereafter. At room temperature, plasma BM levels remained constant for 48 h but fell to 50% of their original values after 8 days.

FIG. 1.—Standard curve for bleomycin.

Human plasma and urine samples, taken following BM therapy, were pooled and stored in 200-μl aliquots at —20°C. Aliquots were included in routine assays. After 8 assays of the plasma pool, inter-assay and within-assay variations were both 12%. After 5 assays of the urine pool, inter-assay variation was 20% and within-assay variation 16%.

The assay results on plasma samples

TABLE II.—*Drugs Exhibiting No Reaction with Antiserum when Present in the Assay in 1-μg Amounts*

| | |
|---|---|
| Methotrexate | Amphetamine |
| Adriamycin | Cocaine |
| Vincristine | Tetracycline |
| Cytosine arabinoside | Promazine |
| Mercaptopurine | Ephedrine |
| Fluorouracil | Nicotine |
| Procarbazine | Trifluoperazine |
| Carbamezepine | Diphenhydramine |
| Phenytoin | Lignocaine |
| Nortriptyline | Digoxin |
| Lysergic acid | Methadone |
| Morphine | Diazepam |
| Codeine | |

FIG. 2.—Plasma levels of bleomycin in 2 rabbits after an i.v. dose of 0·5 mg/kg given at time 0.

collected from rabbits following i.v. doses of 0·5 and 1·0 mg/kg are shown in Figs. 2 and 3. Each of the 4 disappearance curves showed two clearance phases over the 3-h collection period. The calculated half-lives for the first and second phases were 9–12 and 30–42 min respectively.

BM concentrations were measured in plasma and urine samples collected from hospital patients undergoing treatment with the drug. The plasma levels in Patient 1, who had received 30 mg BM i.v., are shown in Fig. 4. They indicate a two-phase clearance with estimated plasma half-lives of 43 and 140 min respectively. Patient 2 received 15 mg of BM i.v. Plasma BM levels and the amount of BM recovered in the urine over the time of specimen collection are shown in Fig. 5. The plasma clearance in this individual showed only a single phase with a half-life of 150 min. Approximately 50%

FIG. 3.—Plasma levels of bleomycin in 2 rabbits after an i.v. dose of 1 mg/kg given at time 0.

FIG. 4.—Plasma bleomycin levels in a patient after an i.v. dose of 30 mg given at time 0.

FIG. 5.—Measurement of plasma and urinary bleomycin levels in a patient after an i.v. dose of 15 mg given at time 0.

of the BM administered was recovered in the urine during the first 7·5 h.

### DISCUSSION

It is apparent from this study and an earlier report by Broughton and Strong (1976) that BM–protein conjugates are highly immunogenic and capable of eliciting high-titre high-avidity antisera in immunized animals. In addition to stimulating the production of antibodies in sheep we have also raised high-titre antisera in rabbits, but these have not been considered in the present paper.

Since the therapeutic preparation of BM is a mixture of $A_1$, $A_2$ and $B_2$ components, antisera were tested for reactivity with the individual pure compounds. HP/S/66-1C exhibited similar avidities for the $A_2$ and $B_2$ components which comprise 99% of the therapeutic mixture. For this reason this antiserum was used in the assay of specimens. Whether the antiserum also cross-reacts with BM metabolites and/or immunoreactive fragments remains speculative, but the similarity in plasma disappearance and urinary excretion rates when measured by RIA (Fig. 4) or bioassay (Ohnuma *et al.*, 1974) suggest that the RIA measures total antibiotic activity, which is probably, though not necessarily, equivalent to its antitumour activity.

The antiserum (HP/S/66-1C) chosen for use in the assay showed no cross-reaction with several therapeutic drugs in common use, nor with other antineoplastic agents often used in combination with BM. The assay specificity for BM, therefore, permits drug measurements to be performed on untreated plasma and urine samples.

The RIA of BM in plasma showed good recovery values when the drug was added to normal plasma and urine. Variation in recovery occurred when specimens were stored at 4°C for prolonged periods or at room temperature for short periods. It has been reported (Ohnuma *et al.*, 1974) that inactivation of BM by plasma can occur during incubation at 37°C, although this treatment had no effect on urinary levels. The sensitivity of the RIA is 8 ng/ml, compared with the reported range of 100–250 ng/ml for bacteriological assay. Although the latter sensitivity range is adequate for the measurement of plasma levels shortly after the usual i.v. dose of BM, the RIA offers a much simplified, more rapid and sensitive procedure. Furthermore, tissue drug levels should be detectable and measurable by the RIA method, but so far we have no information on this point.

The application of RIA to the measurement of BM levels during therapy should provide a rapid method for continuous control of patient dosage and should be especially useful when applied to combination therapy in which BM is administered together with other cytotoxic drugs such as methotrexate (Aherne, Piall and Marks, in preparation) and vincristine (Teale, Clough and Marks, 1977) for which RIAs have been produced.

We are grateful to the Cancer Research Campaign for financial support. Specimens from patients were kindly supplied by Dr W. White and his staff at St Luke's Hospital, Guildford.

### REFERENCES

ALBANO, J. & EKINS, R. P. (1970) The Attainment of High Sensitivity and Precision in Radioimmunoassay Techniques as Exemplified in a Simple Assay of Serum Insulin. In: *In Vitro Procedures with Radioisotopes in Medicine.* Vienna: I.A.E.A. p. 491.

BROUGHTON, A. & STRONG, J. E. (1976) Radioimmunoassay of Bleomycin. *Cancer Res.*, **36**, 1418.

COHEN, S. S. & I., J. (1976) Synthesis and the Lethality of Bleomycin in Bacteria. *Cancer Res.*, **36**, 2768.

OHNUMA, T., HOLLAND, J. F., MASUDA, H., WALIGUNDA, J. A. & GOLDBERG, G. A. (1974) Microbiological Assay of Bleomycin: Inactivation, Tissue Distribution, and Clearance. *Cancer, N.Y.*, **33**, 1230.

TEALE, J. D., FORMAN, E. J., KING, L. J., PIALL, E. M. & MARKS, V. (1975) The Development of a Radioimmunoassay for Cannabinoids in Blood and Urine. *J. Pharm. Pharmac.*, **27**, 465.

TEALE, J. D., CLOUGH, J. M. & MARKS, V. (1977) Radioimmunoassay of Vinblastine and Vincristine. *Br. J. clin. Pharmac.*, **4**, 169.

UMEZAWA, H. (1973) Studies on Bleomycin: Chemistry and the Biological Action. *Biomedicine*, **18**, 459.